# Impact of daily prompt to vaccinate inpatients awaiting rehabilitation against SARS-CoV-2 and influenza

**DOI:** 10.1017/ice.2024.221

**Published:** 2025-02

**Authors:** Amber Linkenheld, Victoria R. Williams, Karen Chan, Helene Carating, Romina Marchesano, Jennifer Do, Sherri Sullivan, Danette Beechinor, William K. Silverstein, Jerome A. Leis

**Affiliations:** 1 Sunnybrook Health Sciences Centre, Toronto, ON, Canada; 2 Centre for Quality Improvement and Patient Safety, University of Toronto, Toronto, ON, Canada; 3 Department of Medicine, University of Toronto, Toronto, ON, Canada

## Abstract

A daily prompt to offer vaccination to inpatients awaiting transfer to rehabilitation resulted in increased SARS-CoV-2 (OR 5.64, 95% CI 3.3–10.15; *P* < 0.001) and influenza (OR 3.80, 95% CI 2.45–6.06; *P* < 0.001) vaccination. Compared to baseline, this intervention was associated with reduced incidence of viral respiratory infection during subsequent admission to rehabilitation.

## Introduction

Post-acute care rehabilitation facilities are at risk of respiratory viral transmission in the context of their patient population and programming that involves mobilization and congregation.^1^ Not only do these infections place rehabilitation patients at increased risk of complications, but outbreaks are highly disruptive to completion of rehabilitation programs.

St. John’s Rehabilitation (SJR) is a 154-bed rehabilitation facility of Sunnybrook Health Sciences Centre in Toronto, Canada, receiving approximately 2500 admissions per year, including cardiac, amputee, stroke, trauma, medical debility, burn, and musculoskeletal patients following acute care hospitalization. Before and throughout the COVID-19 pandemic, we instituted a number of measures to mitigate infection risk at SJR, including improvements to staff vaccination rates, surveillance, and testing.^1,2^ Vaccination of patients upon admission to rehabilitation was also implemented but given average length of stay of 25 days, many vaccinated patients remained susceptible for the majority of their admission.

We hypothesized that a standardized prompt to vaccinate hospitalized patients against both SARS-CoV-2 and influenza in advance of their transfer to rehabilitation could improve vaccination coverage and reduce risk of viral respiratory infection during rehabilitation. We undertook this quality improvement study introducing a new vaccine program targeting patients designated as alternate level of care (ALC), defined as those recovered from their acute medical illness and awaiting transfer to post-acute care facilities.^3^

## Methods

At baseline, receipt of vaccinations against SARS-CoV-2 and influenza were routinely recorded on admission by pharmacy technicians as part of best possible medication history. These vaccines were available to inpatients but seldom ordered by clinical teams. At the start of the 2023–2024 viral respiratory season, we implemented a new standardized approach to vaccination of all acute care inpatients upon designation as ALC, consistent with local and national guidelines.^4–6^ An automated daily report was created of ALC patients without documented receipt of either SARS-CoV-2 (XBB.1.5-adapated) or seasonal influenza (ie, Fluzone Quadrivalent (IIV4-SD), Fluzone High dose Quadrivalent (IIV4-HD) or Fluad (IIV3-Adj)) upon admission to hospital. Eligible patients were communicated daily (except weekends) by Infection Preventionists by secure e-mail to the unit-level pharmacist, most responsible physician, charge nurse, and patient care manager to assess, consent and administer vaccination as appropriate to these target inpatients. The daily report of ALC patients eligible for vaccination was made available during daily interprofessional clinical rounds.

We performed a before-after study assessing vaccination rates during baseline (2022–23) and intervention (2023–24) seasons throughout identical periods (10 October to 19 January). The primary outcome was the proportion of ALC patients vaccinated before discharge, defined as receipt of seasonal SARS-CoV-2 or influenza vaccine during hospital stay. For comparison, community-initiated vaccination was measured at baseline for SARS-CoV-2 and during intervention for both influenza and SARS-CoV-2, based on recorded receipt of seasonal vaccines on admission. Due to variability in documentation, community-based influenza vaccination was not available at baseline.

The secondary outcome was the incidence of healthcare-associated (HA) SARS-CoV-2 and influenza (onset >72 hours after admission) in rehabilitation patients, with a subgroup analysis among those vaccinated greater than 14 days prior. Proportions and incidence adjusted per 1000 patient days were compared using the χ^2^ test, odds ratio and 95% confidence intervals. Research ethics board approval was obtained.

## Results

There were 747 and 859 ALC patients during baseline and intervention periods, respectively. Age (72 vs 73 years), gender (50.2 vs 47.4% male), and proportion admitted to general internal medicine (48.6 vs 50.8%) were similar between both periods. A similar majority of ALC patients were transferred to SJR during both seasons (57.5 vs 63.9%) with the remainder transferred to other rehabilitation, alternate care facilities, or to the community.

The proportion of ALC patients vaccinated before discharge increased for both SARS-CoV-2 (2.0% vs 10.4%; OR 5.64, 95% CI 3.3–10.15; *P* < 0.001) and influenza (3.3% vs 11.6%; OR 3.80, 95% CI 2.45–6.06; *P* < 0.001). The rate of community-based SARS-CoV-2 vaccination also increased, to a smaller degree (7.8% vs 10.9%; OR 1.46, 95%CI 1.04–2.07; *P* = 0.02).

**Figure 1.**
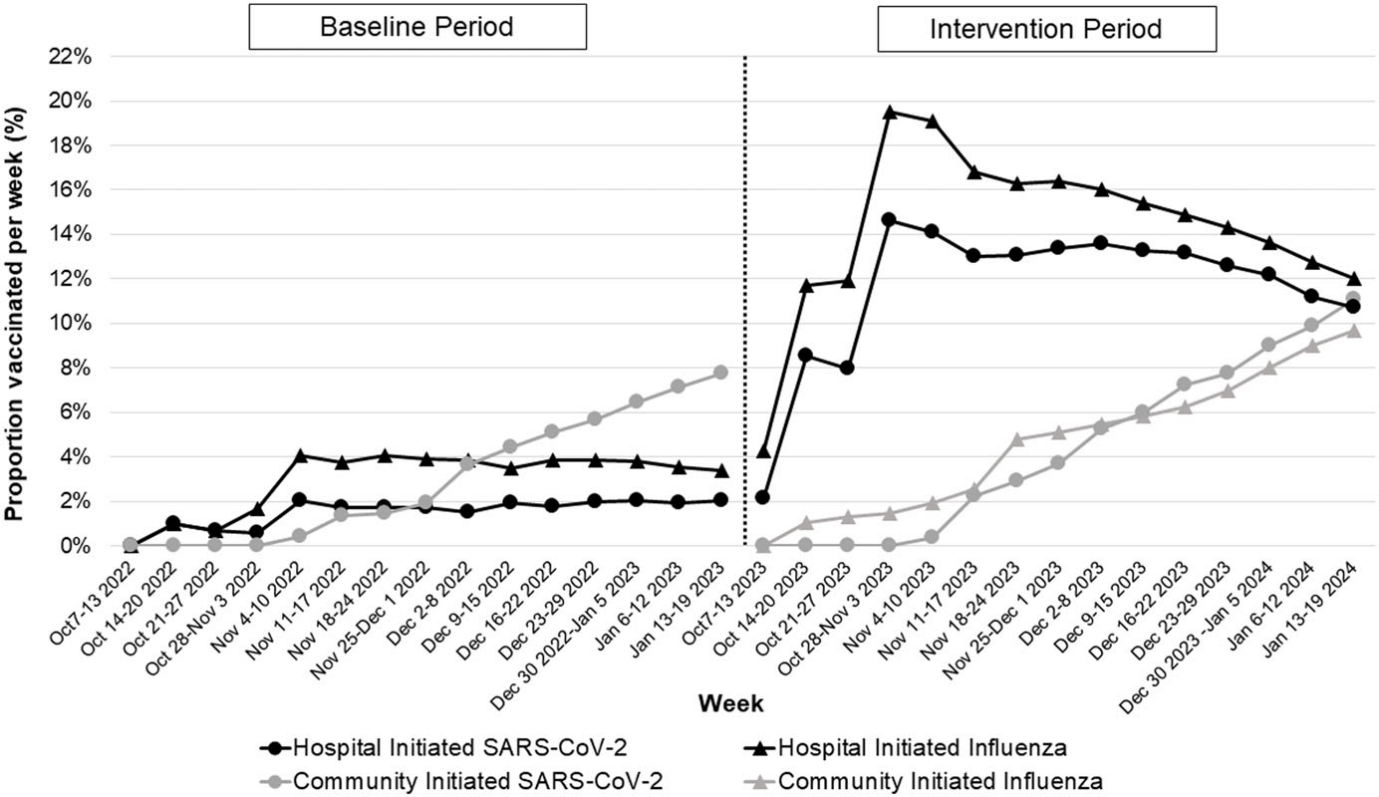
Cumulative proportion of weekly patients designated as alternate level of care (ALC) who were vaccinated against SARS-CoV-2 or influenza by discharge, stratified by community versus hospital-initiated vaccination.

Figure 1 illustrates the cumulative weekly proportion of ALC patients that received either hospital-initiated versus community-initiated vaccination between both seasons. During the intervention period, hospital-initiated vaccination against SARS-CoV-2 and influenza contributed to 78.4% and 76.3% of vaccination in October–November, compared to 29.4% (*P* < 0.001) and 33.7% (*P* < 0.001) in December–January.

The incidence of HA-SARS-CoV-2 in rehabilitation decreased during the intervention season compared to baseline (73 vs 110 cases; 2.09 vs 3.24 per 1000 pt days; OR 0.64, 95% CI 0.48–0.87; *P* = 0.004) with no significant difference for HA-influenza given small numbers (0 vs 3 cases). In the subgroup analysis, vaccinated ALC patients during the intervention season experienced a lower risk of HA-SARS-CoV-2 compared to those unvaccinated (2.5%, 2/81 vs 10.6%, 52/490, *P* = 0.02). The same subgroup analysis for HA-influenza identified 0 cases among vaccinated ALC patients compared to 3 among those remaining unvaccinated. Combined risk of either SARS-CoV-2 or influenza among those vaccinated was significantly lower (OR 0.2, 95% CI 0.03–0.71; *P* = 0.01).

## Discussion

We found that a daily prompt to vaccinate ALC patients against both SARS-CoV-2 and influenza significantly improved vaccination coverage of this target group. Those vaccinated prior to discharge had an 80% lower odds of viral respiratory infection during subsequent stay in rehabilitation.

Prior studies demonstrated the safety of in-hospital influenza vaccination, with no overall increase in fever or risk of re-admission, which supports this practice.^7^ Despite this, a 2021 review of hospital-based vaccination initiatives identified challenges in implementation as many clinicians may consider vaccine delivery to be the responsibility of primary care.^8^ The same review suggested that multi-component strategies were the most effective including standardizing the approach with reminders.

Our daily prompt was aligned with this approach and applied it to a specific target group of patients that were most likely to benefit given their documented increased risk of HA-viral respiratory infection during rehabilitation.^5,6^ Though vaccine uptake of our intervention did not reach optimal rates, they did exceed national uptake for SARS-CoV-2 vaccination during the 2023–2024 season.^9^ The intervention could be enhanced further with the introduction of additional behavioral change interventions.^10^ Embedding daily prompts into rounds may have facilitated uptake but also contributed to increased workload in a busy clinical environment. Given the lower return in the later phase of the season due to the most amenable patients having already been vaccinated in the community, the time lines of this intervention could be restricted to improve efficiency.

Our study has significant limitations. First, this is an uncontrolled before-after study that is subject to confounding factors. Rates of HA-infection may have been influenced by differences in seasonal incidence and community-based vaccination. Despite this, our subgroup analysis suggests that those vaccinated as part of this intervention had reduced risk of this outcome. Second, we did not measure rehabilitation vaccination rates which could have influenced these outcomes, although no change was made to this process and these vaccines would have conferred limited protection based on the average length of stay. Third, rehabilitation facilities typically accept patients across many different hospitals which would require a similar intervention to be implemented across all acute care facilities to optimize impact.

Vaccination of acute care inpatients awaiting transfer may reduce the risk of viral respiratory infection during rehabilitation. A standardized approach to vaccinating this target group could be scaled across hospitals to maximize impact annually, especially between October and December.
